# Supplementary material for: Intracellular Conformation of Amyotrophic Lateral Sclerosis-Causative TDP-43
Source: Int J Mol Sci. 2023 Mar 14;24(6):5513. doi: 10.3390/ijms24065513 (PMC10056606; doi:10.3390/ijms24065513)
Supplement: Supplementary file 1 [file ijms-24-05513-s001.zip › ijms-2272905-supplementary.pdf]

Supplementary Materials

Intracellular conformation of amyotrophic lateral sclerosis-causative TDP-43

Akira Kitamura [1,*], Sachiko Yuno [2], Rintaro Kawamura [3], and Masataka Kinjo [4]

[1] Laboratory of Molecular Cell Dynamics, Faculty of Advanced Life Science, Hokkaido University; PRIME, Japan Agency for Medical Research and Development

[2] Laboratory of Molecular Cell Dynamics, Graduate School of Life Science, Hokkaido University

[3] Laboratory of Molecular Cell Dynamics, Graduate School of Life Science, Hokkaido University

[4] Laboratory of Molecular Cell Dynamics, Faculty of Advanced Life Science, Hokkaido University

* Correspondence: akita@sci.hokudai.ac.jp; Tel.: +81(11)-706-9006

Figure S1

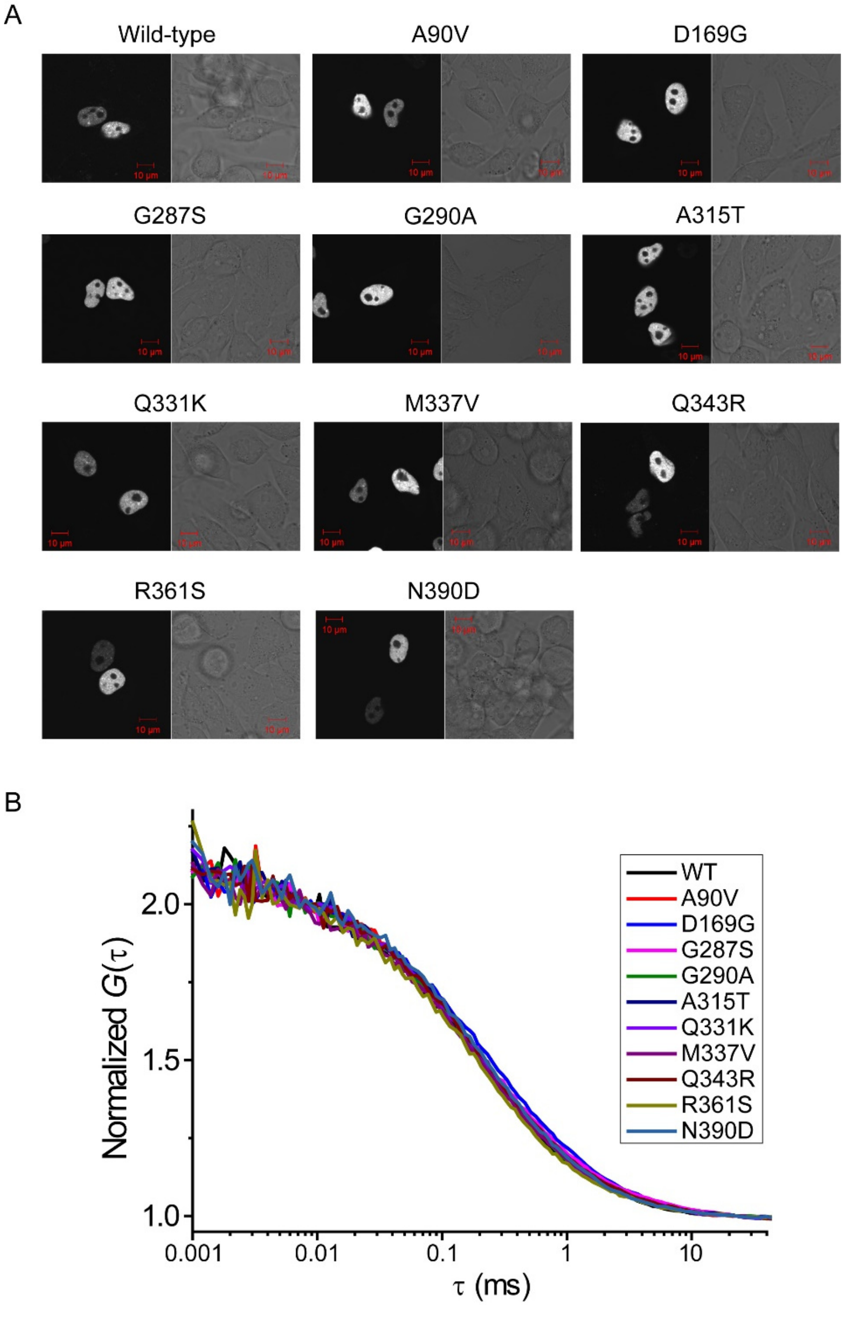

Figure S1 Subcellular localization and diffusion state of ALS-associated mutants of TDP-43 tagged with eGFP.

(A) Confocal fluorescence images of eGFP-labeled wild-type and ALS-associated mutants of TDP-43. Alphabets and numbers indicate the one-letter symbol of the amino acid containing the mutation and the location of the amino acid residue. Bars = 10 μm. (B) Normalized autocorrelation functions of eGFP-labeled wild-type (WT) and ALS-associated mutants of TDP-43 in cell lysates. No significant differences in the autocorrelation curve were observed.
